# Pre-treatment CEMRI habitat radiomics and deep learning for noninvasive prediction of the VETC pattern in hepatocellular carcinoma: an exploratory radiogenomic analysis

**DOI:** 10.3389/fonc.2026.1809603

**Published:** 2026-06-26

**Authors:** Xingwu Xie, Yanxi Xiong, Xiao-Shan Huang, Xiaojuan Tang, Yue Zhao, Xiaoyu Xiao, Long Jin

**Affiliations:** 1 Department of Interventional Radiology, Beijing Friendship Hospital, Capital Medical University, Beijing, China; 2 Department of Interventional Radiology, Renmin Hospital, Hubei University of Medicine, Shiyan, Hubei, China; 3 Department of Radiological Imaging Center, Renmin Hospital, Hubei University of Medicine, Shiyan, Hubei, China; 4 Department of Radiology, The Second Affiliated Hospital of Zhejiang Chinese Medical University, Hangzhou, China

**Keywords:** DL, hepatocellular carcinoma, intratumoral heterogeneity, ITH, MRI, TCGA, VETC

## Abstract

**Background:**

Vessels encapsulating tumor clusters (VETC) constitute an aggressive morphological subtype of hepatocellular carcinoma (HCC), characterized by marked angiogenesis and immune infiltration, and can potentially be identified noninvasively using radiomics.

**Objective:**

This study aimed to evaluate the predictive performance of pre-treatment contrast-enhanced MRI (CEMRI) habitat radiomics and deep learning models for identifying VETC in HCC, and to characterize the associated immune infiltration patterns.

**Methods:**

We retrospectively analyzed CEMRI scans from 336 patients with HCC (336 lesions). Habitat features and deep learning (DL) features were extracted from the CEMRI scans, and the LASSO was applied for feature selection to construct an intratumoral heterogeneity (ITH) model and a deep learning (DL) model. Robust ITH and DL features were subsequently integrated to develop a fusion model with enhanced predictive capability, and model performance was assessed using the area under the receiver operating characteristic curve (AUC). Using radiomics features derived from the fusion model, 47 HCC patients with baseline liver MRI scans in The Cancer Imaging Archive (TCIA) and matched RNA-seq profiles in The Cancer Genome Atlas (TCGA) were stratified into a high-risk group (n = 23) and a low-risk group (n = 24). Transcriptomic profiles were then analyzed using gene set variation analysis (GSVA) to compare immune-related pathway activity between groups. Differential gene expression analysis (DGEA), gene set enrichment analysis (GSEA), and cell type identification by estimating relative subsets of RNA transcripts (CIBERSORT)-based immune infiltration analysis were further performed to elucidate potential differences in the tumor immune microenvironment and their biological implications.

**Results:**

The ITH model achieved AUCs of 0.845 and 0.806 in the training and validation cohorts, respectively, whereas the DL model yielded AUCs of 0.764 and 0.745. The fusion model demonstrated superior performance, with AUCs of 0.901 and 0.870 in the respective cohorts. Transcriptomic analyses identified significant differential gene expression between the high- and low-risk groups. GSEA indicated that the low-risk group was enriched in pathways related to the cell cycle, translation, and mitochondrial function. Immune profiling revealed a significant reduction in resting dendritic cells in the high-risk group (*P* < 0.05), suggesting potential immune evasion.

**Conclusions:**

A CEMRI-based fusion model integrating ITH and DL features enables accurate prediction of VETC in HCC and shows preliminary associations with immune-related transcriptomic and infiltration profiles linked to this vascular pattern.

## Introduction

HCC represents a major global health burden and is the third leading cause of cancer-related mortality worldwide. Despite advances in curative approaches, including hepatectomy and liver transplantation, the prognosis of patients with HCC remains poor (1, 2). The high recurrence rate and restricted long-term survival underscore the inherently aggressive nature of HCC. Within the established framework of HCC metastasis, epithelial-to-mesenchymal transition (EMT) has long been recognized as a classical route to tumor dissemination (3). In recent years, a newly characterized vascular pattern—VETC—which facilitates HCC metastasis independently of EMT, has gained considerable attention as a distinct histologic subtype. This pattern is defined by small tumor cell clusters encased by endothelial cells, giving rise to multiple discrete, spheroid-like units. These units can readily merge with microvessels and subsequently enter the circulatory system. Encapsulation by endothelial cells enables these clusters to evade immune surveillance while in circulation. Upon reaching distant organs, these clusters are released into the target tissue, where they colonize, proliferate, and ultimately establish metastatic lesions. This EMT-independent route of dissemination is referred to as the VETC metastatic pattern in HCC (3). VETC has been strongly linked to aggressive metastatic behavior, early postoperative recurrence, and unfavorable survival outcomes (4). Accurate recognition of VETC-positive tumors is essential for optimizing clinical decision-making, including surgical margin assessment and planning of adjuvant therapies. Although pathological biopsy remains the gold standard for diagnosing VETC (5), its invasive nature, time-consuming workflow, and susceptibility to sampling errors arising from tumor heterogeneity highlight the need for reliable, noninvasive preoperative assessment. Habitat radiomics, which partitions tumors into voxel-level subregions to quantify intratumoral heterogeneity (ITH), offers a promising approach to address this challenge (6, 7). These subregions reflect intrinsic biological properties and the tumor microenvironment, providing valuable insights into tumor behavior and aggressiveness (8–10). Although habitat-based radiomics has been applied in several malignancies, such as breast and esophageal cancer, its use in predicting VETC in HCC based on CEMRI remains limited. In parallel, deep learning (DL) has emerged as a major advance in radiomics, enabling high-dimensional feature extraction through sophisticated convolutional neural network (CNN) architectures (11). DL models have demonstrated strong performance in predicting pathological characteristics and clinical outcomes across multiple cancer types. CEMRI, with its superior soft-tissue contrast and ability to capture heterogeneous vascular patterns across multiple phases, represents an ideal modality for both radiomics and DL analyses and is particularly suitable for identifying imaging correlates of VETC (12). However, no prior study has integrated CEMRI-derived habitat radiomics with DL features to develop a robust predictive model for VETC.

The aim of this study was to assess and compare the ability of the CEMRI-based habitat radiomics ITH model and the DL model to predict the VETC pattern and to determine their clinical utility. In addition, we integrated ITH and DL features to develop a fusion model that enables noninvasive, pre-treatment prediction of VETC. Transcriptomic data from HCC patients in The TCGA were further incorporated to explore underlying immune-infiltration patterns and to provide a quantitative tool to support clinical decision-making.

## Materials and methods

### Patient cohort and clinical data

This study was conducted in accordance with the Declaration of Helsinki and was approved by the Ethics Committee of our Hospital, which granted a waiver of informed consent (Approved in March 2025 No: SYRMYY-2025-060). Patients with histologically confirmed HCC who underwent evaluation between January 2018 and May 2025 were retrospectively enrolled. The inclusion criteria were as follows:(a) histopathological confirmation of HCC based on surgical resection or biopsy specimens;(b) availability of CEMRI performed within two weeks prior to surgery;(c) complete clinical data, including age, sex, etiology (HBV, HCV, or other causes), alpha-fetoprotein (AFP), alanine aminotransferase (ALT), aspartate aminotransferase (AST), prothrombin time (PT), albumin level, and Child–Pugh classification.

**Figure 1:**
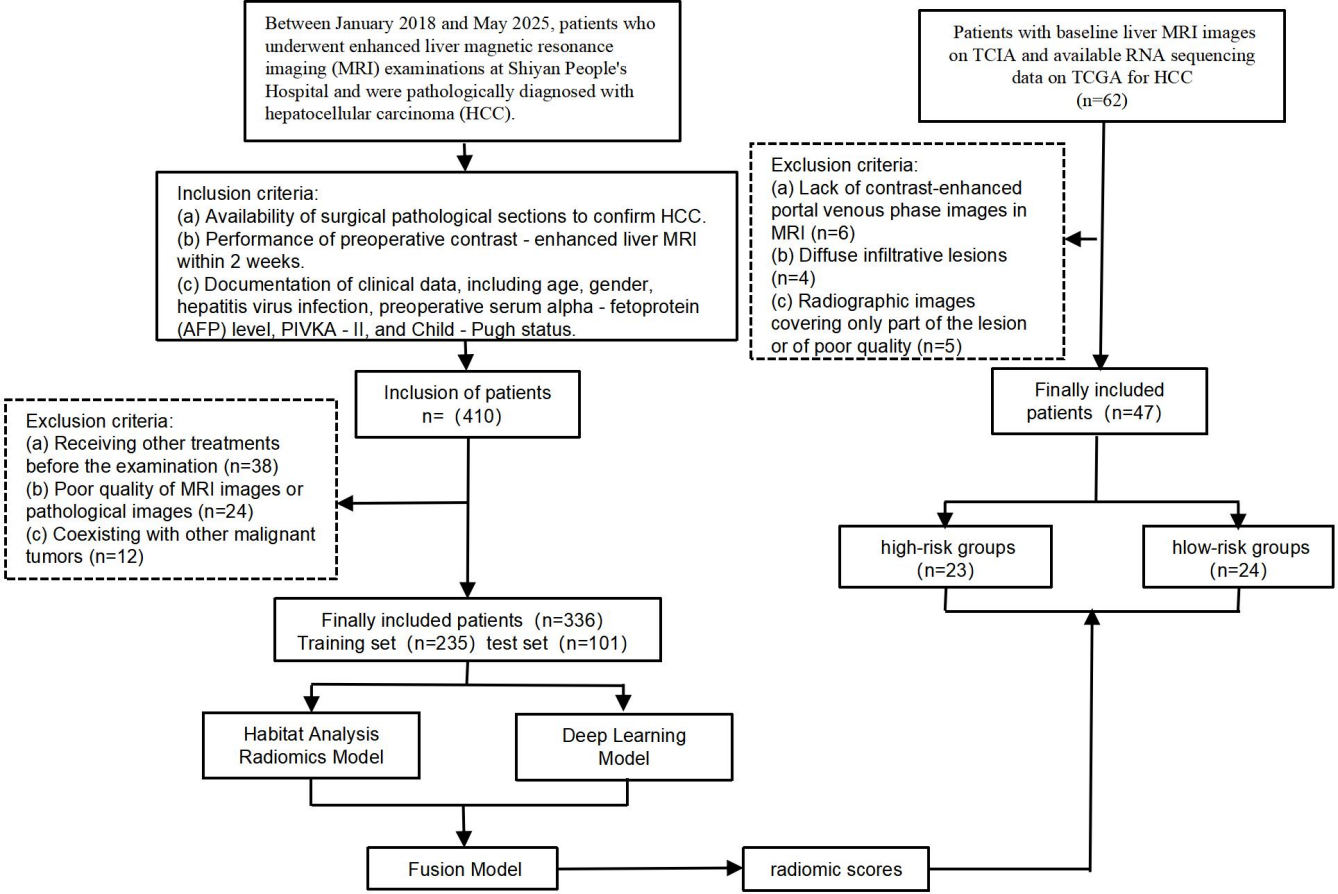
Study flowchart of patient inclusion and exclusion. CEMRI, contrast-enhanced MRI; HCC, hepatocellular carcinoma; VETC, vessels encapsulating tumor clusters.

The exclusion criteria were:(a) receipt of any treatment before MRI acquisition (n = 38);(b) suboptimal MRI or pathological image quality (n = 24);(c) presence of concurrent malignancies (n = 12). After applying these criteria, 336 patients with HCC were included in the final cohort. The study workflow is illustrated in **Figure 1**.

### Histopathological assessment of VETC and tumor differentiation

Liver tissue samples were subjected to CD34 immunohistochemical staining and independently reviewed by two hepatopathologists with more than five years of diagnostic experience. The reviewers were blinded to all preoperative imaging findings, and any discrepancies were adjudicated by consensus. Tumors were classified as VETC-positive when these structures constituted at least 50% of the entire tumor area (for surgical specimens) or 50% of the evaluable tumor tissue (for biopsy specimens). This 50% threshold falls within the 50%-55% prognostic gold-standard interval established by high-quality evidence. A 2024 systematic review and meta-analysis by Wang et al. (5), which included 13 studies and 4,429 HCC patients, demonstrated that thresholds in this range have the highest prognostic value, significantly outperforming lower thresholds (1% or any presence). This interval includes the 55% cutoff used in the landmark study by Renne et al. (4) and the 50% cutoff used in multiple other high-quality studies. Two hepatopathologists independently reviewed all slides, and discrepancies were resolved by consensus. The interobserver agreement for VETC diagnosis was excellent (Cohen’s kappa (κ) = 0.89). Tumor differentiation was graded as well-, moderately-, or poorly differentiated HCC. When heterogeneous differentiation patterns were present, the predominant subtype was used for the final diagnosis.

### MRI scanning protocol and image preprocessing

MRI examinations were performed using two 3.0-T scanners (Lumina and Skyra, Siemens Healthineers, Germany). The MRI protocol consisted of the following sequences: (1) T1-weighted imaging (T1WI); (2) T2-weighted imaging (T2WI); (3) diffusion-weighted imaging (DWI); and (4) CEMRI. A gadolinium-based contrast agent (Gd-DTPA; gadopentetate dimeglumine, Germany) was intravenously administered at a dose of 0.2 mL/kg and an injection rate of 1 mL/s. A three-dimensional volume-interpolated breath-hold examination (VIBE) combined with bolus tracking was used to acquire arterial phase (AP, 25–35 seconds), portal venous phase (PVP, 60–70 seconds), and delayed phase (DP, 180 seconds) images. The overall acquisition time for CEMRI was approximately 3–4 minutes.

Preprocessing procedures were applied prior to region-of-interest (ROI) segmentation:

(1) N4 bias-field correction to mitigate intensity nonuniformity caused by scanner hardware and magnetic field inhomogeneities; (2) voxel resampling using linear interpolation to standardize voxel spacing to 1×1×1 mm³ across all scans; (3) z-score intensity normalization to standardize signal intensity distributions to a mean of 0 and standard deviation of 1, eliminating systematic intensity differences between the two scanners. These preprocessing steps were implemented to standardize the imaging data and ensure comparability across different scanners and acquisition protocols for subsequent analyses.

### ROI delineation

ROI delineation was performed on preprocessed portal venous phase (PVP) images. While arterial phase is the primary sequence for HCC diagnosis, our study focused on quantifying intratumoral vascular heterogeneity (the core of habitat analysis) and predicting the VETC pattern. PVP was selected because it avoids the uniform hyperenhancement that obscures subtle vascular differences between intratumoral subregions, and achieves the highest tumor-liver contrast-to-noise ratio, enabling accurate segmentation with excellent interobserver agreement (ICC = 0.89) in our cohort. This approach is consistent with published evidence supporting PVP as the optimal phase for HCC habitat radiomics (13) and VETC prediction (14).

ROI delineation was performed on the preprocessed portal venous phase images by a radiologist with 5 years of experience in abdominal imaging diagnosis using the open-source software ITK-SNAP (version 3.6.0; www.itk-snap.org). The tumor margins were manually traced on consecutive axial slices to generate a three-dimensional ROI encompassing the entire lesion. All manual segmentations were reviewed and confirmed by two experienced radiologists, each with 10 years of expertise in abdominal imaging, to ensure accuracy. To assess interobserver and intraobserver agreement, 30 cases were randomly selected and re-segmented by both radiologists with a one-week interval between sessions. For patients with multifocal HCC, only the largest lesion was selected for segmentation. These procedures were implemented to ensure the consistency and reliability of ROI segmentation for subsequent analyses.

### Habitat-based feature extraction

The entire tumor ROI was imported into the PyRadiomics package (version 3.1), from which a comprehensive set of radiomics features was extracted. These included first-order features (n=18), shape descriptors (n=14), and texture features derived from the gray level co-occurrence matrix (GLCM) (n=24), gray level dependence matrix (GLDM) (n=14), gray level run length matrix (GLRLM) (n=16), gray level size zone matrix (GLSZM) (n=16), and neighborhood gray tone difference matrix (NGTDM) (n=5) matrices. A k-means clustering algorithm was applied to partition intratumoral voxels into subregions with similar enhancement characteristics. The number of clusters (k) was varied from 2 to 10, and the optimal k was determined exclusively in the training cohort (n=235) using the Calinski–Harabasz (CH) score, with the highest CH value indicating the best cluster separation. The optimal k value was identified as 3, corresponding to three distinct functional subregions with different intratumoral blood supply. After clustering, voxels belonging to the same habitat were assigned identical color labels to generate a spatial habitat distribution map on MRI images. Ultimately, the CEMRI-derived habitats yielded a total of 963 (321 × 3) habitat features per HCC lesion.

### Deep learning feature extraction

Deep learning (DL) features were extracted using the ResNet50 convolutional neural network pretrained on the ImageNet dataset. Prior to feature extraction, preprocessing included selecting and cropping the slice with the largest tumor ROI and normalizing voxel intensities to the range [0, 1] via min–max scaling. To tailor the model to the prediction task, ResNet50 was fine-tuned by optimizing network weights with a cross-entropy loss function. The adaptive moment estimation optimizer was applied with a learning rate of 0.1. The network was trained for 500 epochs with a batch size of 50, after which the parameters were fixed for feature extraction. For each HCC lesion, a total of 2048 DL features were derived from the penultimate layer (average pooling layer) of the fine-tuned ResNet50 across all CEMRI phases. Dimensionality reduction was performed using principal component analysis (PCA) to mitigate overfitting. Furthermore, Gradient-weighted Class Activation Mapping (Grad-CAM) was employed to visualize feature importance, providing intuitive heat-map representations of the contribution of different tumor regions to the model’s predictive output.

### Feature dimensionality reduction and construction of quantitative scores

**Figure 2:**
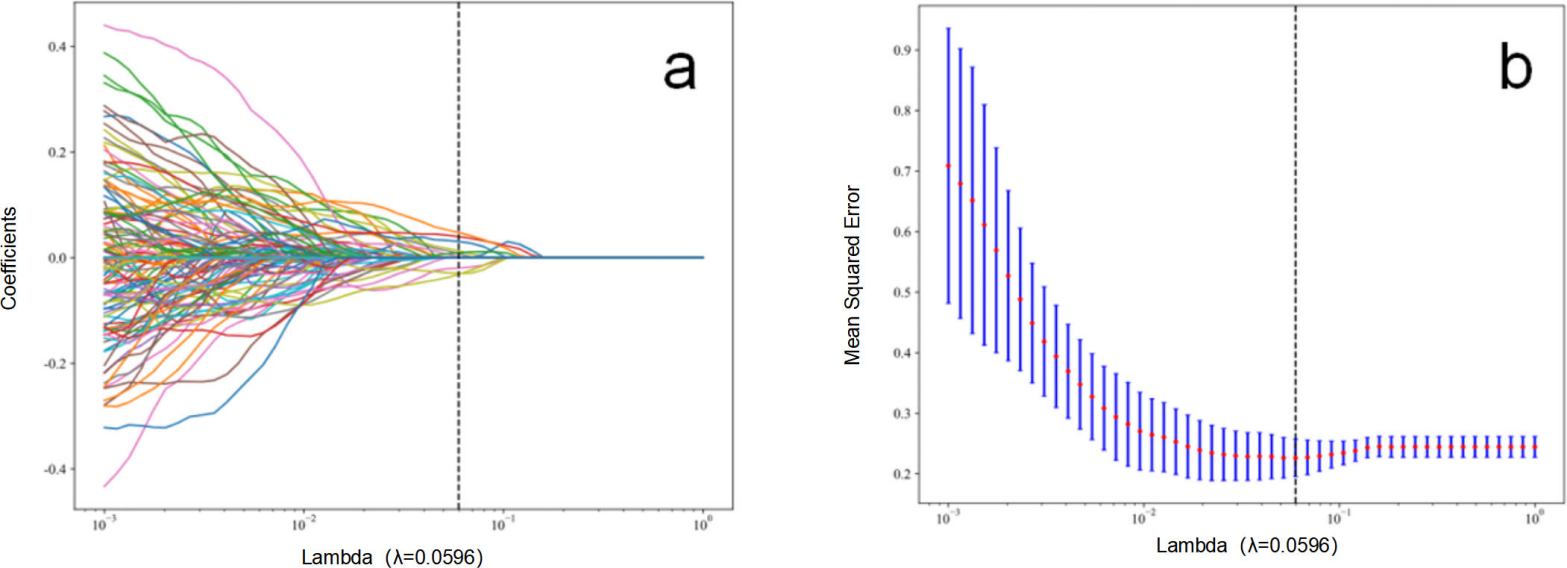
LASSO-based selection of radiomic features. **(A)** LASSO coefficient profiles of features in the training cohort. The dashed line indicates the λ value determined by 10-fold cross-validation, and each curve represents the trajectory of an individual predictor. **(B)** Ten-fold cross-validation plot for dimensionality reduction using the LASSO method in the training cohort. The vertical dashed line marks the optimal Log(λ) value selected according to the minimum criterion.

A four-step pipeline was implemented to reduce feature dimensionality and identify stable predictors. First, all features derived from habitat analysis and CEMRI sequences underwent Z-score normalization. Second, Spearman rank correlation analysis was used to remove highly collinear features (correlation coefficient > 0.9). Third, LASSO regression with 10-fold cross-validation was performed to select features with non-zero coefficients based on the optimal penalty parameter (λ). Finally, the minimum redundancy maximum relevance (mRMR) algorithm was applied to retain the most relevant features while minimizing redundancy. Using the refined feature set, a logistic regression model was developed. The regression coefficients served as feature weights to compute quantitative scores for predicting VETC status and tumor differentiation. Three scoring systems were generated: the ITH model score, the DL model score, and the integrated ITH + DL score. **Figure 2** illustrates the LASSO-based selection of radiomic features.

### Development of predictive models and assessment of clinical utility

**Figure 3:**
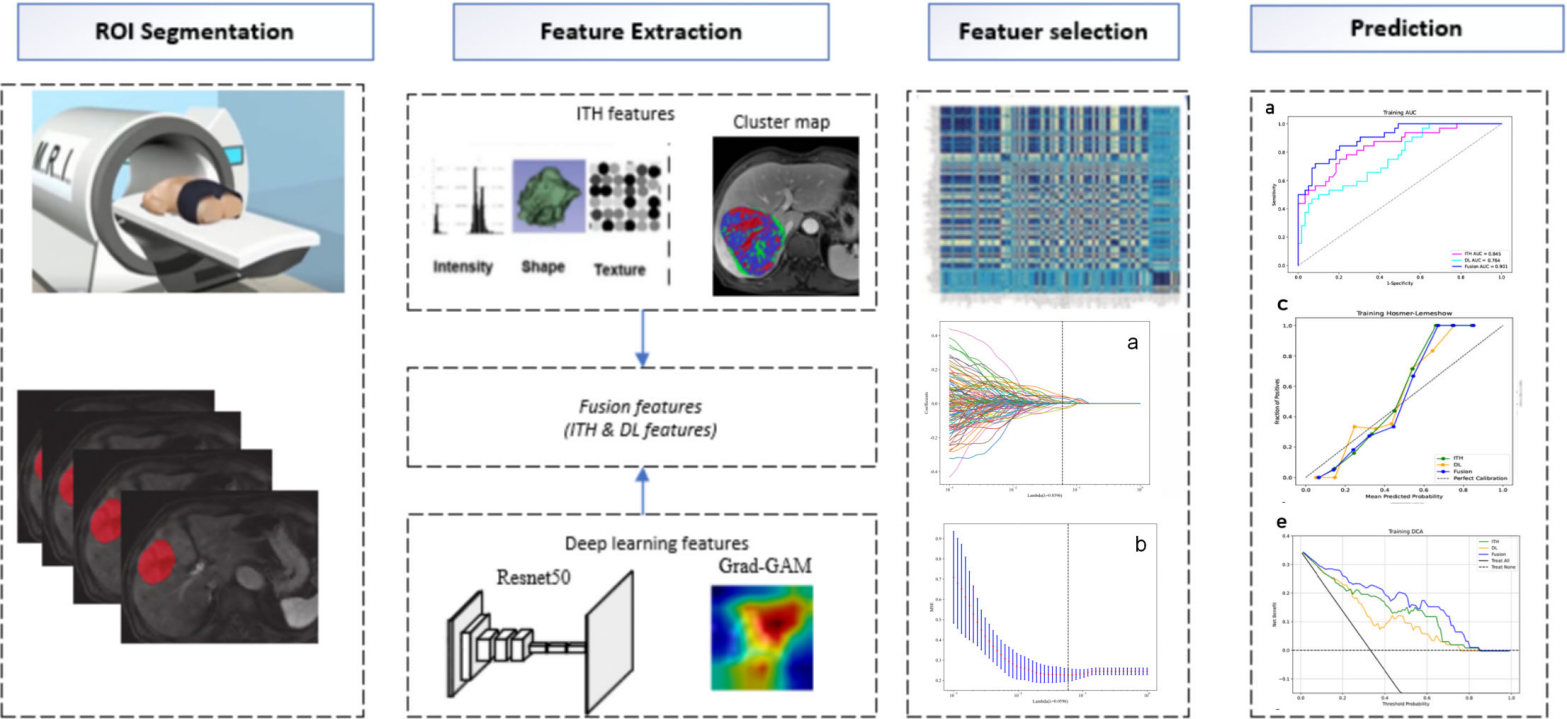
Workflow from CEMRI acquisition to final model construction. Pre-processed portal-venous-phase CEMRI images were used for (i) habitat-based intratumoral-heterogeneity (ITH) feature extraction, (ii) ResNet50-based deep-learning (DL) feature extraction, and (iii) fusion-model development integrating ITH and DL features. ROI, region of interest; PCA, principal component analysis; SVM, support vector machine; AUC, area under the curve.

In this study, three predictive models were developed to estimate VETC status in both the training and validation cohorts:(1) an ITH model constructed using habitat-based radiomic features;(2) a DL model derived from deep-learning features;(3) a fusion model integrating both ITH and DL features. All models were built using a Support Vector Machine (SVM) classifier. Calibration performance was assessed using the Hosmer–Lemeshow goodness-of-fit test, and clinical utility was evaluated by decision curve analysis (DCA). Across both cohorts, the fusion model consistently demonstrated superior predictive performance compared with the ITH and DL models. **Figure 3** illustrates the complete workflow from CEMRI acquisition to final model construction.

### Exploratory radiogenomic and immune analyses in the TCIA/TCGA matched cohort

To elucidate the molecular context of the radiomics signature, we performed an exploratory radiogenomic analysis in a TCIA/TCGA matched cohort (n = 47), in which baseline liver MRI was available from TCIA and corresponding RNA-seq profiles were available from TCGA. Importantly, we transferred the fusion-model imaging signature trained and finalized in our institutional CEMRI cohort and applied it directly to the TCIA MRI data to compute the radiomics risk score (Rad-score) for each TCIA case; no re-training, feature re-selection, or parameter tuning was performed in the public cohort. Patients were then dichotomized into high- and low-risk groups using the median Rad-score of the training cohort (median = 0.42, interquartile range: 0.28–0.57). This cutoff was determined exclusively in the training cohort and applied unchanged to the TCIA external cohort to avoid data leakage.

Differential expression analysis was conducted using the limma package (|log2 fold change (log2FC)| > 1, false discovery rate (FDR) < 0.05), followed by GSVA and GSEA to characterize pathway-level differences between groups. In addition, immune cell proportions were inferred using CIBERSORT and group differences were compared.

### Statistical analysis

All statistical analyses were conducted using R software (version 4.0). Continuous variables are presented as medians with interquartile ranges (IQR), and categorical variables as counts and percentages. Differences between the training and validation cohorts were evaluated using the Kruskal-Wallis H test for continuous variables and the χ² test for categorical variables. Intraclass correlation coefficient (ICC) were calculated to assess interobserver and intraobserver variability in ROI segmentation and feature extraction. The diagnostic performance of the models for predicting VETC status and pathological differentiation was assessed by calculating the area under the receiver operating characteristic (ROC) curve (AUC), sensitivity, specificity, and accuracy. Differences in AUC between models were compared using the DeLong test. Differences in subregion distributions between VETC-positive and VETC-negative groups were evaluated using the Mann-Whitney U test. All statistical tests were two-tailed, and *P* < 0.05 was considered statistically significant.

## Results

### Patient characteristics

A total of 336 patients with pathologically confirmed HCC were included, comprising 114 VETC-positive cases (33.93%) and 222 VETC-negative cases (66.07%). Notably, the positivity rate was significantly higher in surgical resection specimens (36.8%) compared to needle biopsy specimens (22.4%, P = 0.032), consistent with the known tendency of biopsy to underestimate VETC status due to sampling error. The interobserver agreement for VETC-positive diagnosis was excellent (κ = 0.89).The patients were randomly assigned to a training cohort (n = 235) and a validation cohort (n = 101) at a 7:3 ratio. The mean ages of patients in the training and validation cohorts were 60.14 ± 10.34 and 61.23 ± 10.22 years, respectively. In the training and validation cohorts, the proportions of male patients were 80.85% and 87.13%, HBV/HCV-related HCC accounted for 69.79% and 66.37%, and 60.00% and 76.24% of patients had AFP levels ≥20 ng/mL, respectively. No significant differences were observed between the two cohorts in liver function indices, Child-Pugh classification, or VETC status (P > 0.05) (**Table 1**).

**Table 1:** Baseline characteristics of included HCC patients.

Variable/feature	Total (n = 336)	Training cohort (n = 235)	Validation cohort (n = 101)	P value
Age (years)	59.15 ± 10.30	60.14 ± 10.34	61.23 ± 10.22	0.77
Sex (n,%)				0.92
Male	278 (82.73)	190 (80.85)	88 (87.13)	
Female	58 (17.27)	45 (19.15)	13 (12.87)	
Specimen type (n,%)				0.68
Specimen type	269 (80.06)	188 (80.00)	81 (80.20)	
Needle biopsy	67 (19.94)	47 (20.00)	20 (19.80)	
Etiology (n,%)				0.41
HBV/HCV	231 (68.75)	164 (69.79)	67 (66.37)	
Others	105 (31.25)	71 (30.21)	34 (33.63)	
AFP (n,%)				0.28
≥20 ng/mL	218 (64.88)	141 (60.00)	77 (76.24)	
<20 ng/mL	118 (35.12)	94 (40.00)	24 (23.76)	
ALT (U/L)	26.93 (18.47–39.70)	26.49 (18.32–39.71)	27.13 (18.78–34.34)	0.83
AST (U/L)	31.84 (25.68–43.75)	31.64 (25.35–42.95)	32.14 (26.51–47.45)	0.66
PT (s)	12.20 (11.60–12.96)	12.11 (11.60–13.02)	12.31 (11.69–12.99)	0.74
Albumin (g/L)	40.37 (37.30–43.80)	40.29 (37.24–43.45)	40.85 (37.70–44.68)	0.35
Child-Pugh grade (n,%)				0.29
A	272 (80.95)	190 (80.85)	82 (81.19)	
B	64 (19.05)	45 (19.15)	19 (18.81)	
VETC (n,%)				0.89
no	222 (66.07)	153 (65.11)	69 (68.32)	
yes	114 (33.93)	82 (34.89)	32 (31.68)	

AFP, alpha-fetoprotein; ALT, alanine aminotransferase; AST, aspartate aminotransferase; HBV, hepatitis B virus; HCV, hepatitis C virus; HCC, hepatocellular carcinoma; PT, prothrombin time; VETC, vascular encapsulated tumor cluster.

### Optimal habitat cluster number determination

**Figure 4:**
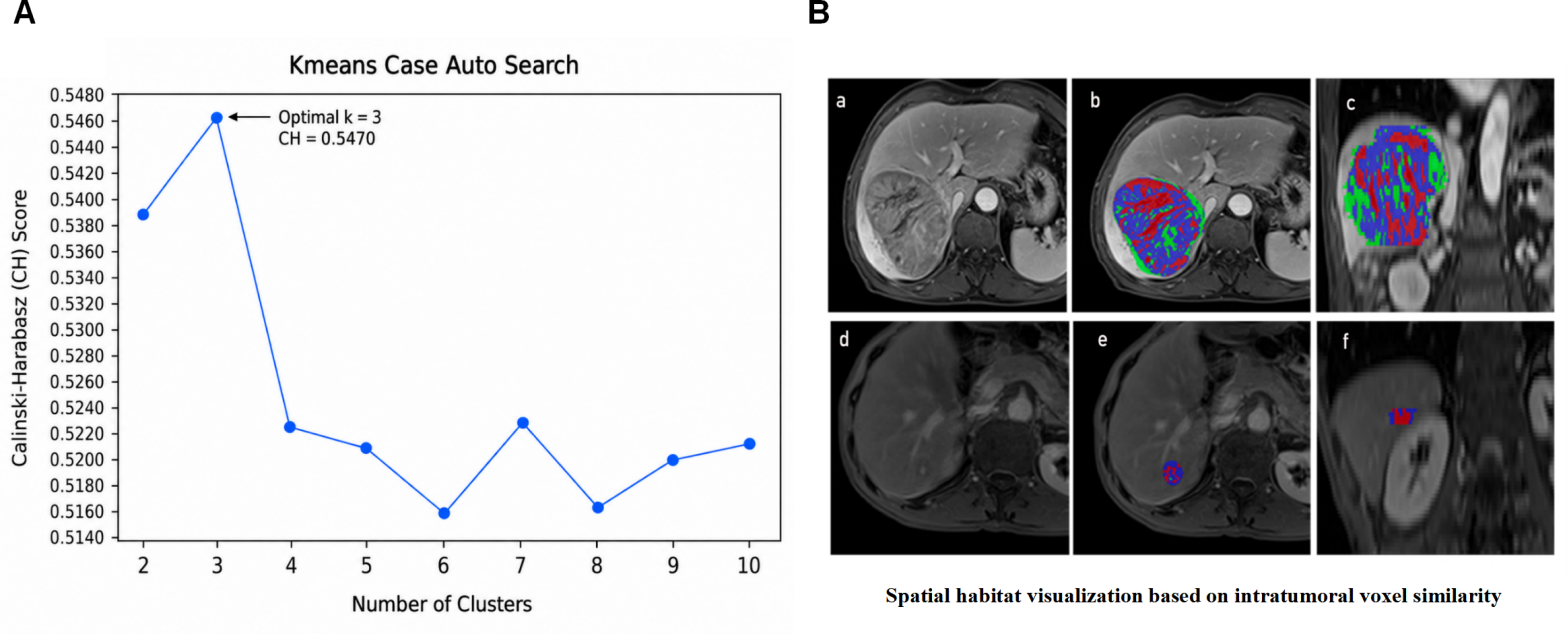
Determination of the optimal number of habitat clusters and representative cases. **(A)** Selection of the optimal cluster number based on the Calinski–Harabasz (CH) score. The CH score curve reaches its peak at k = 3 (CH = 0.5470), indicating that three intratumoral subregions represent the optimal partition scheme for capturing tumor heterogeneity. **(B)** Spatial habitat visualization based on intratumoral voxel similarity. (A–C), A 62-year-old male patient with VETC-positive HCC. The tumor predominantly consisted of high blood supply regions (red subregions) and medium blood supply regions (green subregions), with only a small fraction of low blood supply regions (blue subregions), suggesting pronounced intratumoral heterogeneity (ITH). Postoperative pathology was consistent with VETC positivity and poor differentiation. (D–F), A 65-year-old male patient with HCC. The lesion predominantly contained low blood supply regions (blue subregions) and high blood supply regions (red subregions), indicating a lower ITH score. Postoperative pathology was consistent with VETC negativity and high differentiation.

The optimal number of habitat clusters was determined in the training cohort using the Calinski–Harabasz (CH) score. As shown in **Figure 4A**, the CH score reached its peak value of 0.5470 at k=3 and gradually decreased with increasing k values, indicating that three subregions provided the best statistical separation of intratumoral heterogeneity. **Figure 4B** presents two representative cases of HCC tumors segmented into three habitat subregions on portal venous phase MRI slices, visualizing the spatial distribution of habitats with different blood perfusion characteristics and their correlation with VETC status.

### Feature selection and quantitative score calculation

Interobserver and intraobserver agreement for subregion features were 0.89 (95% confidence interval (CI): 0.83–0.97) and 0.88 (95% CI: 0.82–0.93), respectively. After removing redundant features through Spearman correlation analysis, a total of 237 habitat analysis features and 94 DL features were retained. Subsequently, nine features were selected using the LASSO algorithm and seven features were selected using the mRMR algorithm to construct the ITH and DL models for predicting VETC-positive HCC, respectively. By integrating the selected ITH and DL features, a fusion model comprising 16 features was developed to predict VETC status. Quantitative scores for each HCC lesion were computed based on the selected features and their regression coefficients. The feature selection process and the feature weights demonstrated the contribution of each feature to the predictive performance of the models.

### VETC prediction and clinical application assessment

**Tables 2**, **3**, along with **Figure 5**, summarize the diagnostic performance of the three models for predicting VETC status. As shown in **Table 2**, the fusion model achieved the highest AUC values of 0.901 (95% CI: 0.86–0.94) in the training cohort and 0.870 (95% CI: 0.85–0.96) in the validation cohort, demonstrating superior performance compared with the other models. The ITH model ranked second, with AUCs of 0.845 (95% CI: 0.82–0.94) and 0.806 (95% CI: 0.77–0.95) in the training and validation cohorts, respectively, whereas the DL model exhibited the lowest performance, with AUCs of 0.764 (95% CI: 0.68–0.85) and 0.745 (95% CI: 0.65–0.88). In the validation cohort, the fusion model also achieved higher sensitivity (0.94) and specificity (0.83) than the ITH and DL models, respectively. The Hosmer-Lemeshow test indicated good calibration for all models, with calibration curves showing near-perfect alignment, particularly for the fusion and ITH models. As shown in **Figure 5**, decision curve analysis (DCA) further demonstrated that the fusion model provided the highest net clinical benefit across a range of threshold probabilities, indicating superior clinical applicability compared with the ITH and DL models. **Table 3** presents pairwise comparisons using DeLong’s test, showing that the differences in AUC between any two models were statistically significant (*p* < 0.05), indicating meaningful differences among the three models. The fusion model had the highest AUC, significantly outperforming the ITH and DL models; the ITH model ranked second and was also significantly better than the DL model. For VETC-positive (n = 114) and VETC-negative (n = 222) HCC lesions, portal venous phase MRI images were segmented to quantify the volumes of subregions with different blood supply levels. The proportion of high-blood-supply subregions (red) was calculated as the High-Blood-Supply Fraction (HBSF), medium-blood-supply subregions (green) as the Medium-Blood-Supply Fraction (MBSF), low-blood-supply subregions (blue) as the Low-Blood-Supply Fraction (LBSF), along with the ITH score for each lesion. As shown in **Table 4**, VETC-positive tumors exhibited a significantly higher proportion of high-blood-supply subregions, indicating more active vascular architecture; the proportion of medium-blood-supply subregions was slightly lower, potentially associated with higher tumor differentiation, while the proportion of low-blood-supply subregions was significantly decreased, suggesting an overall higher blood supply in VETC-positive HCC. **Figure 4** highlights differential subregions within HCC lesions associated with varying VETC status and pathological differentiation, further elucidating the relationship between ITH and clinical outcome.

**Table 2:** Diagnostic performance of models for predicting VETC status.

Model	Number of features	Cohort	Accuracy	AUC	95% CI	Sensitivity	Specificity
DL	7	Training	0.76	0.764	0.68–0.85	0.76	0.78
		Validation	0.74	0.745	0.65–0.88	0.79	0.74
ITH	9	Training	0.86	0.845	0.82–0.94	0.83	0.85
		Validation	0.83	0.806	0.77–0.95	0.81	0.79
Fusion	16	Training	0.89	0.901	0.86–0.94	0.89	0.82
		Validation	0.85	0.870	0.85–0.93	0.94	0.83

**Table 3:** Pairwise comparisons of AUC between models using DeLong test.

Model comparison	AUC	95% CI	Z value	*P* value
DL VS ITH	0.081	0.68–0.85	10.43	<0.001
DL VS Fusion	0.137	0.82–0.94	19.25	<0.001
ITH VS Fusion	0.055	0.92–0.97	8.77	<0.001

DL,Deep Learning; ITH,Intratumoral Heterogeneity Model; VETC,Vascular Encapsulated Tumor Cluster; Fusion,Fusion Model (DL + ITH).*Z* values indicate the test statistic used to assess the significance of differences between AUCs; larger *Z* values correspond to greater differences. All *Z* values > 1.96 correspond to *P* < 0.05.

**Figure 5:**
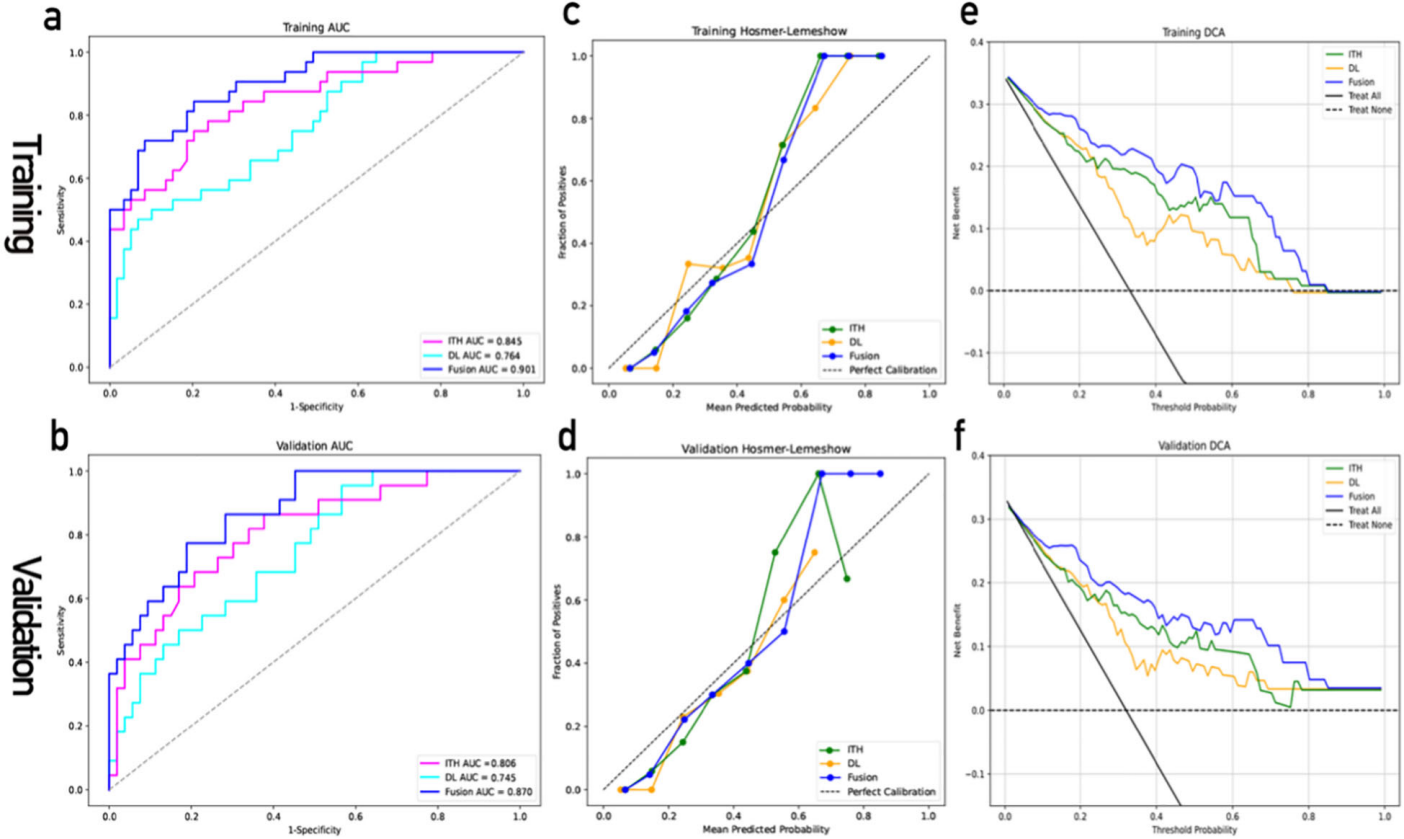
Performance of the three models for predicting VETC status. **(A, B)** ROC curves show AUCs for the training **(A)** and validation **(B)** cohorts. The fusion model achieved the highest AUCs: 0.901 (95% CI: 0.86–0.94) and 0.870 (95% CI: 0.85–0.93), respectively. **(C, D)** Calibration curves demonstrate good agreement between predicted and observed probabilities. **(E, F)** Decision curve analysis (DCA) indicates that the fusion model provides the highest net clinical benefit across a range of threshold probabilities. AUC, area under the curve; CI, confidence interval; DCA, decision curve analysis; ITH, intratumoral heterogeneity; DL, deep learning; VETC, vessels encapsulating tumor clusters.

**Table 4:** Comparison of tumor subregion fractions between VETC-positive and VETC-negative HCC lesions.

Parameter	VETC-positive(n=114)	VETC-negative(n=222)	Difference between groups (95% CI)	*P* value
HBSF(%)	58.0 ± 9.0	52.0 ± 9.0	6.0(2.2-9.8)	0.004
MBSF(%)	26.5 ± 5.0	29.5 ± 5.0	-3.0(-5.8 to -0.2)	0.039
LBSF(%)	15.5 ± 4.0	18.5 ± 4.0	-3.0(-5.7 to -0.2)	0.020
ITH score	0.55 ± 0.12	0.45 ± 0.12	0.10(0.02-0.18)	0.015

HBSF, MBSF, and LBSF denote the high-, medium-, and low-blood-supply subregions, respectively. The percentage distributions of all subregions differed significantly between the VETC-positive and VETC-negative groups (*P < 0.05*).

### Preliminary External Validation in the TCIA Cohort

To assess the generalizability of our fusion model, we performed preliminary external validation on 32 HCC patients from the TCIA/TCGA matched cohort with available pathological VETC assessment. Our final fusion model, which was trained and finalized exclusively in our institutional cohort, was applied directly to the TCIA MRI data without any retraining or parameter adjustment. The model achieved an AUC of 0.792 (95% CI: 0.63-0.91) in this independent cohort, with a sensitivity of 0.75 and specificity of 0.82. The slightly lower performance compared to our institutional validation cohort (AUC = 0.870) may be attributed to differences in MRI scanning protocols, patient demographics, and pathological assessment practices across institutions. These preliminary results support the potential generalizability of our model, although further validation in larger, standardized multicenter cohorts is warranted.

### Immune infiltration patterns associated with the radiomics model

**Figure 6:**
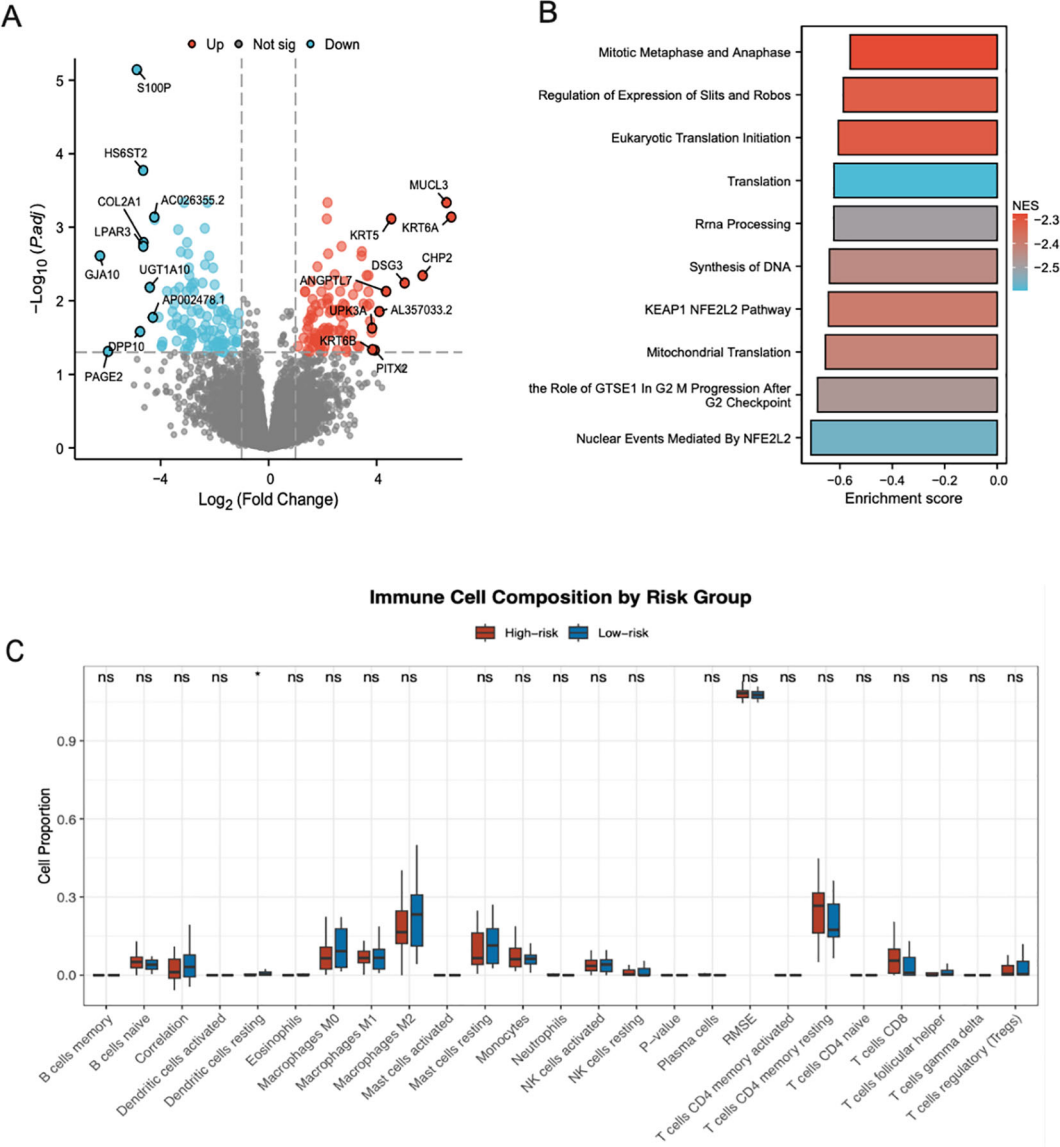
Bioinformatics analysis based on 47 hepatocellular carcinoma (HCC) patients with MRI images from the TCIA dataset. **(A)** Volcano plot showing differentially expressed genes (DEGs) between high-risk and low-risk groups (|log_2_FC| > 1 and FDR < 0.05). Upregulated genes in the high-risk group are shown in red, while downregulated genes are shown in blue. **(B)** Gene set enrichment analysis (GSEA) of the top 10 most significantly downregulated pathways (normalized enrichment score, NES < 0). Enriched pathways in the low-risk group include those related to cell cycle, translation, and mitochondrial functions. **(C)** Immune cell infiltration landscape estimated using CIBERSORT. Boxplots compare immune cell proportions between high-risk and low-risk groups. Asterisks indicate statistical significance (*p* < 0.05); “ns” denotes non-significant differences.

Based on the radiomics risk score (Rad-score) computed by applying our fusion-model signature to TCIA baseline liver MRI, the TCIA/TCGA matched cohort (n = 47) was stratified into Rad-score–defined high-risk (n = 23) and low-risk (n = 24) groups. Differential expression results are summarized in **Figure 6A**. The volcano plot illustrates significant transcriptomic differences between groups (|log_2_FC| > 1, FDR < 0.05), with genes upregulated in the high-risk group shown in red and downregulated genes in blue, suggesting that imaging-based stratification is accompanied by distinct molecular profiles. As shown in **Figure 6B**, GSEA indicated that pathways related to cell-cycle regulation, translation, and mitochondrial function were enriched in the low-risk group compared with the high-risk group (corresponding depletion in the high-risk group; normalized enrichment score (NES) < 0). Immune infiltration analysis using CIBERSORT (22 immune cell types) is presented in **Figure 6C**. Most immune cell fractions were not significantly different between groups, whereas resting dendritic cells were reduced in the high-risk group (P < 0.05). Together, these findings provide preliminary evidence that the radiomics-defined imaging phenotype may be associated with differences in immune microenvironment, warranting confirmation in larger cohorts.

## Discussion

This study developed and validated three CEMRI-based predictive models—namely, the ITH model, DL model, and Fusion model—for the noninvasive assessment of VETC patterns in HCC patients. Among them, the Fusion model, which integrates habitat radiomics and deep learning features, achieved AUCs of 0.901 and 0.870 in the training and validation cohorts, respectively, significantly outperforming models based solely on habitat or deep learning features. To the best of our knowledge, this study presents three key innovations: (1) it is the first to integrate CEMRI-based habitat radiomics with deep learning features for noninvasive VETC prediction in HCC; (2) it is the first to establish a quantitative link between habitat blood supply subregions, VETC pathological patterns, and the tumor immune microenvironment; (3) it addresses the common limitation of poor interpretability in conventional whole-tumor radiomics by combining radiogenomic analysis to provide biological insights into VETC-associated imaging phenotypes. These findings highlight the potential of the Fusion model for preoperative identification of VETC-positive HCC and for informing individualized therapeutic strategies.

In recent years, radiomics and deep learning approaches have been increasingly applied to predict aggressive tumor characteristics and treatment outcomes in HCC (15, 16). Most prior studies have focused on using MRI semantic features or conventional whole-tumor radiomics to predict microvascular invasion and tumor differentiation, demonstrating satisfactory predictive performance (17–19). In addition, several studies have indicated that certain imaging features on contrast-enhanced CT and EOB-MRI—such as intratumoral necrosis on enhanced CT, heterogeneous arterial phase enhancement on EOB-MRI, and tumor-to-liver signal ratio ≤ 0.585 during the hepatobiliary phase—may be predictive of VETC status (20, 21). Notably, Hu et al. performed EOB-MRI-based radiomics analyses, constructing intratumoral, peritumoral, and combined radiomics models to predict VETC. They demonstrated that the peritumoral radiomics model could noninvasively identify VETC-positive HCC, and that VETC-positive patients were more prone to early postoperative recurrence and shorter progression-free survival (14). Collectively, these studies underscore the value of imaging in VETC prediction. However, most remain within the conventional “whole-tumor average information” radiomics paradigm, primarily emphasizing risk stratification while providing limited insight into the invasive biology and immune microenvironment of VETC.

Our study proposed a CEMRI-based strategy that integrates habitat analysis with deep learning features. This approach not only enhanced VETC prediction performance but also preliminarily revealed intratumoral vascular–immune phenotypes associated with VETC, thereby partially addressing the common limitation of “predictive but difficult to interpret” in prior research. Furthermore, prior deep learning studies (12) (e.g., Chu et al.) applied convolutional neural networks on dynamic contrast-enhanced MRI to simultaneously predict microvascular invasion and VETC patterns, demonstrating the potential of DL for extracting high-dimensional imaging information. Nevertheless, single whole-tumor CNN models have two notable limitations. First, directly inputting the entire tumor into the network hinders discrimination of subregions with distinct blood supply and cellular density, potentially reducing sensitivity to critical heterogeneous regions. Second, these models lack interpretability linked to tumor biological features, limiting mechanistic insights. In contrast, our study addressed these issues in three ways: (i) habitat analysis segmented different blood supply subregions at the voxel level, enabling spatial quantification of ITH; (ii) integration of habitat and DL features markedly improved diagnostic performance and clinical net benefit; and (iii) combined TCIA/TCGA transcriptomic data were used to explore immune infiltration and functional pathways corresponding to radiomics risk stratification, providing potential biological explanations for VETC imaging phenotypes.

As a radiomics-based approach, habitat analysis clusters similar voxels within the tumor, refining the conventional “whole-tumor average features” into multiple subregions with distinct perfusion and histological significance, thus facilitating quantitative characterization of ITH (22–27). Compared with whole-tumor radiomics, this method reduces interference from necrotic, hemorrhagic, and cystic regions and better captures critical regions associated with tumor invasiveness. Notably, quantitative analysis of high-, medium-, and low-blood-supply habitats revealed that VETC-positive tumors exhibited a significantly higher proportion of high-blood-supply subregions, a markedly lower proportion of low-blood-supply subregions, and relatively higher ITH scores, consistent with the pathological characteristic of VETC as a highly perfused vascular configuration prone to hematogenous metastasis.

In this study, 2048 deep learning (DL) features were extracted from the penultimate fully connected layer of the fine-tuned ResNet50 model. To reduce the risk of overfitting, principal component analysis (PCA) was applied to these features to generate a more manageable feature set. Notably, although the DL model demonstrated good discriminative ability in both the training and validation sets, its performance in predicting VETC was slightly inferior to the ITH model based on habitat analysis. This phenomenon suggests that relying solely on CNNs to extract deep features from the whole lesion may be insufficiently sensitive to VETC patterns with complex spatial heterogeneity, whereas voxel-wise subregion quantification based on habitat analysis is more effective in capturing VETC-related feature variations (11). On this basis, this study integrated these two complementary feature types, and the resulting Fusion model outperformed either single model in terms of AUC, sensitivity, specificity, and decision curve analysis, further emphasizing the necessity of combining multi-level features to improve diagnostic performance.

These findings are consistent with prior studies showing that integrating radiomics and deep learning can improve predictive performance (28–30). For example, Huang et al. (30) combined multiparametric MRI radiomics features with deep learning features to predict response to neoadjuvant chemotherapy in breast cancer, demonstrating that the fusion model outperformed either single feature set alone. Building upon this, our study further incorporated habitat analysis and integrated imaging features with transcriptomic data. Through an exploratory radiogenomic analysis, we enhanced the biological interpretability of the radiomics signature, thereby advancing along the “prediction-to-interpretation” workflow. Specifically, we applied the established CEMRI fusion model to the TCIA cohort and stratified HCC patients into high- and low-risk groups according to the radiomics risk score. Transcriptomic analyses revealed distinct gene-expression profiles between the two groups. GSVA and GSEA further suggested that the low-risk group was enriched in pathways related to cell-cycle regulation, translation, and mitochondrial function, whereas these pathways were comparatively attenuated in the high-risk group, potentially reflecting metabolic reprogramming and cellular functional imbalance in VETC-associated tumors. In addition, CIBERSORT-based immune deconvolution showed a significantly lower proportion of resting dendritic cells in the high-risk group (P < 0.05), suggesting that this imaging phenotype may be associated with reduced antigen-presentation capacity and impaired immune surveillance, and providing a potential immunological context for the more aggressive behavior and earlier recurrence tendency observed in VETC-positive HCC. Importantly, we observed concordant signals between imaging-based risk stratification and transcriptomic/immune-infiltration differences in the public matched cohort, providing preliminary evidence for the biological relevance of the imaging phenotype. However, these associations remain primarily correlative and do not support causal inference; further studies in larger, standardized cohorts with paired imaging–pathology/multi-omics data, together with experimental validation, are warranted to confirm reproducibility and to clarify underlying mechanisms.

A notable limitation of current VETC-related research is the absence of unified diagnostic criteria, with reported positivity rates varying widely from 15% to 45% among published studies. The 50% area threshold adopted in the present study is within the 50%–55% prognostic gold-standard range supported by high-quality evidence (13, 14). The relatively higher overall VETC positivity rate of 33.93% in our cohort can be explained by two main factors: (1) the cohort was dominated by surgical specimens (80%), and the positivity rate was markedly lower at 22.4% in the biopsy subgroup; (2) our study included a larger proportion of bulky and more aggressive tumors relative to several previous cohorts. These results demonstrate that specimen type and tumor characteristics exert substantial effects on VETC assessment. Future multicenter studies are warranted to develop a consensus VETC grading system combining morphological features and quantitative cut-offs, and to validate these criteria across diverse specimen types and patient populations.

Despite the novelty and potential clinical utility of our findings, several limitations should be acknowledged. First, this study was limited to contrast-enhanced MRI portal venous phase (PVP) images and did not integrate multi-phase or multi-modal sequences (e.g., arterial phase, delayed phase, DWI). Although our analysis demonstrated that PVP provided the best predictive performance among single-phase models for VETC prediction, future studies could develop multi-modal fusion models that integrate complementary information from all contrast-enhanced phases and functional sequences to capture a more comprehensive view of tumor hemodynamics and further improve model accuracy and generalizability. Second, this is a retrospective single-center study, and cases with incomplete pathological or imaging data were excluded, which may introduce selection bias. Although we performed preliminary external validation in the TCIA cohort and obtained encouraging results (AUC = 0.792), the sample size was small and imaging protocols were not standardized. A large-scale, multicenter prospective validation study is actively being planned to further validate the robustness and generalizability of our model. Third, our model integrates multiple feature extraction stages, which carries an inherent risk of overfitting. To mitigate this risk, all model development steps (habitat clustering, feature selection, deep learning fine-tuning, and classifier training) were performed exclusively in the training cohort, with the validation and external cohorts completely held out. This strict separation of datasets ensures that our performance estimates are unbiased.

Taken together, the CEMRI-based fusion model integrating habitat radiomics and deep learning enables accurate, noninvasive prediction of the VETC pattern in HCC. The accompanying exploratory transcriptomic analyses provide preliminary biological context, suggesting potential links between the imaging-defined phenotype and immune- and metabolism-related programs. Future studies should validate the model in independent, multicenter cohorts with standardized imaging protocols and assess whether multimodal imaging and multi-omics integration can further improve robustness and clinical utility.

## Data Availability

The datasets presented in this study can be found in online repositories. The names of the repository/repositories and accession number(s) can be found in the article/supplementary material.
